# The “WHY” behind pear pollen tube growth

**DOI:** 10.1093/plphys/kiaf592

**Published:** 2025-11-15

**Authors:** Nilesh D Gawande

**Affiliations:** Assistant Features Editor, Plant Physiology, American Society of Plant Biologists; Department of Biotechnology, School of Sciences, Woxsen University, Hyderabad, Telangana 502345, India

In many flowering plants, both the male and female gametes are produced near each other to facilitate self-pollination. However, self-pollination reduces fitness of the offspring. Some plants have evolved a mechanism where self-pollen is recognized and rejected, preventing self-pollination and effectively promoting outcrossing. One such mechanism is S-RNAse-based self-incompatibility (SI), where plants use highly polymorphic regions, known as the S-locus, to regulate pollination (Qiao et al. 2004; Takayama and Isogai 2005). Two tightly linked polymorphic genes that encode S-RNase and SLF (S-locus F-box protein) or SFB (S haplotype-specific F-box protein), located at the S-locus, control the pistil and pollen SI specificity, respectively (Xue 2025). The variants of the S-locus are referred to as haplotypes. If the haplotype of pollen matches one of the two haplotypes of the diploid pistil, the pollen is recognized as self-pollen, resulting in inhibition of pollen tube growth. In contrast, pollen that has a haplotype different from the pistil is recognized as non-self pollen, resulting in pollen tube growth through the style to achieve fertilization.

The WHIRLY (WHY) is a small family of DNA-binding proteins with a characteristic “whirligig” secondary structure and a conserved KGKAAL DNA-binding domain found in angiosperms (Taylor et al. 2022). WHIRLY proteins form tetrameric complexes with melted double-stranded DNA, with each unit binding to a symmetrical inverted repeat sequence on one strand. This arrangement gives the tetramers a whirligig shape (Krause et al. 2009). Most plant species have two WHY proteins, WHY1 and WHY2, of which WHY1 is localized to the nucleus and chloroplast, whereas WHY2 is localized to mitochondria, plastids, and nuclei. The third WHY protein, WHY3, is specific to Arabidopsis and is localized to the chloroplast. The compartmentation of the WHY proteins is critical for their functions in plant growth, development, and defense. However, cellular localizations of WHY1, WHY2, or WHY3 can change, and these proteins can move between compartments in response to metabolic or environmental stress. The WHY proteins are synthesized in the cytosol and transported to the mitochondria and chloroplasts using targeting signals (Taylor et al. 2022).

In a recent study published in *Plant Physiology*, Xu et al. (2025) investigated the role of the pear WHY2 (PbrWHY2) in S-RNase–based gametophytic self-incompatibility (GSI). First, the authors expressed four S-RNases in *Escherichia coli*, which were derived from two pear cultivars: “Dangshansuli” (S-genotype S7S34) and “Huanghua” (S-genotype S1S2). The purified recombinant S-RNase proteins were tested on in vitro cultured pollen tubes of the “Dangshansuli” cultivar. The authors found that self S-RNases exposed pollen tubes were shorter than non-self S-RNases at different time points. Differential expression analysis after a short exposure time for compatible and incompatible pollen tubes revealed that six candidate genes were differentially expressed, including the WHIRLY transcription factor Pbr008042.1 (PbrWHY2).

PbrWHY2 fused with full-length GFP was localized to the nucleus and mitochondria. The authors further carried out the functional validation of WHY2 in pollen tube growth by using T-DNA insertion Arabidopsis *atwhy2* mutants. Homozygous *atwhy2* mutant had shorter pollen tubes, reduced cellulose content, and reduced ROS in pollen tube apices compared to wild type. Silencing *PbrWHY2* with the antisense oligodeoxynucleotide (As-ODN) resulted in impaired pollen tube elongation, while overexpressing *PbrWHY2* under the pollen-specific *Lat52* promoter in the *atwhy2* mutant background restored phenotypes to wild type. These results suggest that PbrWHY2 positively regulates pollen tube growth by enhancing ROS production and cellulose accumulation in the pollen tube walls (Figure).

**Figure.**
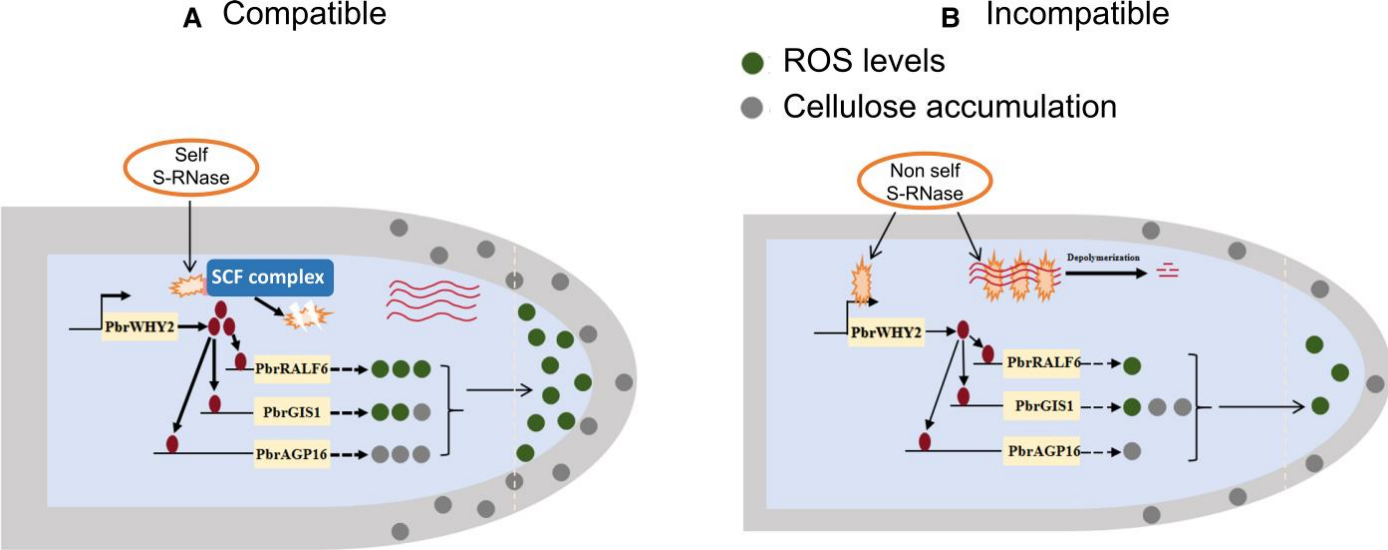
**A)** In compatible pollen tubes, S-RNase is degraded by an SCF (SKP1/Cullin1/F-box) complex, keeping *PbrWHY2* expression stable, which maintains the levels of the other proteins, ROS levels, and cellulose accumulation, promoting pollen tube growth. **B)** In incompatible pollen tubes, self S-RNase suppresses the expression of *PbrWHY2*, which lowers *PbrAGP6*, *PbrRALF6*, and *PbrGIS1* expression levels. This leads to disruption in signaling, compromising cellulose accumulation and reducing ROS levels at the pollen tube tip, ultimately inhibiting tube growth. Figure was adapted from Xu et al. (2025) with few changes.

The authors further investigated how PbrWHY2 affects pollen tube growth when exposed to self-inhibiting S-RNases. The in vitro cultured pollen tubes treated with self S-RNases were shorter than those treated with non-self S-RNases, demonstrating that self S-RNases inhibit pollen tube growth. However, pollen tubes that overexpress PbrWHY2 were longer than wild type when exposed to self S-RNase, indicating that PbrWHY2 reduces the negative effects of self S-RNases, although some inhibition still occurs. The authors further determined the cellulose content of the pollen tube wall and found that *PbrWHY2* overexpression increased cellulose levels in pollen tube walls, though these levels were lower than those found in pollen tubes treated with non-self S-RNases.

*PbrWHY2* overexpression in pollen tubes increased cellulose levels, though it was still lower than the tubes treated with non-self S-RNases, suggesting that PbrWHY2 increases the cellulose accumulation but does not fully prevent its degradation by self S-RNase. The nitroblue tetrazolium test staining assays showed that pollen tubes overexpressing *PbrWHY2* exhibited increased ROS levels compared to wild type. Together, these results demonstrated that PbrWHY2 reduces the effect of self-S-RNases on pollen tube growth, cellulose levels, and ROS accumulation during self incompatibility (SI) in pears.

DNA affinity purification sequencing results showed that PbrWHY2 has a large number of binding peaks in promoter regions, with the GTTAATGA enriched motif. DNA affinity purification sequencing and transcriptome analysis further identified 3 genes, *PbrRALF6* (*RAPID ALKALIZATION FACTOR 6*), *PbrAGP16* (*ARABINOGALACTAN PROTEIN 16*), and *PbrGIS1* (*GENE INVOLVED IN SI REACTION*), which were differentially expressed in compatible and incompatible pollen tubes and consisted of PbrWHY2 binding sites. Silencing or overexpressing *PbrWHY2* altered the expression levels of these genes. Dual luciferase assays, yeast 1-hybrid, and electrophoretic mobility shift assays further confirmed that PbrWHY2 is a transcriptional activator of these genes and binds to the GTTAATGA motif in the promoter regions.

Interestingly, PbrAGP16-GFP and PbrRALF6-GFP fusions were localized to the plasma membrane. It is known that PbrAGP16 is involved in pollen tube development (Li et al. 2018). Silencing *PbrAGP16* led to shorter pollen tubes and reduced cellulose content. PbrRALF2 is known to activate the MAP kinase PbrMPK18, subsequently increasing ROS levels and reducing pollen tube growth. Silencing *PbrRALF6* also resulted in shorter pollen tubes with reduced ROS accumulation at the tube apices, while overexpressing *PbrRALF6* had the opposite effect. Together, these results suggest that PbrAGP16 promotes tube elongation through cellulose accumulation in pollen tube walls and PbrRALF6 promotes pollen tube growth by enhancing ROS levels in pollen tubes (Figure).

PbrGIS1, whose functions are not known, is in the nucleus and cytoplasm. Knockdown of *PbrGIS1* led to reduced pollen tube elongation, decreased ROS accumulation, and increasd cellulose levels, whereas overexpressing *PbrGIS1* had the opposite effect. These results suggest that PbrGIS1 positively regulates pollen tube growth through the modulation of ROS levels in the pollen tip and cellulose levels in the pollen tube walls.

The authors further confirmed the role of WHY2 in regulating *AGP16* and *RALF6* expression during pollen tube growth across species with S-RNase–based GSI by silencing *WHY2* in species such as apple, plum, and pomelo. Silencing WHY2 also resulted in shorter pollen tubes in these species and reduced the expression of *AGP16*. Overexpression of *WHY2* in these species had the opposite effect. These results indicate that only the regulatory functions of WHY2 and AGP16 are conserved across species, whereas RALF6 is not.

Self S-RNase treatment inhibited pollen tube growth in apple and pomelo at different time points. Moreover, self S-RNase reduced the expression of *WHY2* and *AGP16* in apple and pomelo, while in plum, the opposite effect was found, suggesting that the response to self S-RNases varies among different species. These results demonstrated that the role of WHY2 to self S-RNases to GSI is not conserved across all species.

## Recent related articles in *Plant Physiology*:

1. Liu et al. (2025) studied the genomic origin and evolution of S-RNase genes in eudicots using large-scale phylogenetic and microsynteny analysis.
2. Xu et al. (2024) explored the self-incompatibility mechanism in pummelo and identify myo-inositol oxygenase (*CgMIOX3*), which interacts with S-RNases and is crucial for pollen tube growth.
3. Zhang et al. (2024) investigated the role of the *BrRBOHF* gene in *Brassica rapa*, which is responsible for ROS production and modulating self-pollen growth.
4. Zhang et al. (2025) identified the protein PbRbohD1 and its interaction with PbrROP2 as key regulators of ROS levels, which regulate pollen tube growth and fruit set rates, suggesting the pathways for improving pollination efficiency in pears.
